# Grain boundary widening controls siderite (FeCO_3_) replacement of limestone (CaCO_3_)

**DOI:** 10.1038/s41598-023-30757-y

**Published:** 2023-03-20

**Authors:** Juliane Weber, Vitalii Starchenko, Jan Ilavsky, Lawrence F. Allard, Jitendra Mata, Lisa Debeer-Schmitt, Carolyn Grace Cooke, Ken Littrell, Lilin He, Rui Zhang, Andrew G. Stack, Lawrence M. Anovitz

**Affiliations:** 1 Chemical Sciences Division, Oak Ridge National Laboratory, MS 6110, Oak Ridge, TN 37830 USA; 2 Argonne National Laboratory, Chicago, USA; 3 Material Science and Technology Division, Oak Ridge National Laboratory, Oak Ridge, TN 37820 USA; 4 Australian Centre for Neutron Scattering (ACNS), Australian Nuclear Science and Technology Organisation (ANSTO), Lucas Heights, NSW 2234 Australia; 5 Oak Ridge National Laboratory, Oak Ridge, TN 37830 USA; 6 Tennessee Technological University, Cookeville, TN 38505 USA; 7 Chemical Sciences Division, Oak Ridge National Laboratory, One Bethel Valley Road, Bldg. 4100, Rm. C348, MS-6110, Oak Ridge, TN 37831-6110 USA

**Keywords:** Environmental sciences, Environmental chemistry, Geochemistry, Mineralogy

## Abstract

The microstructure of minerals and rocks can significantly alter reaction rates. This study focuses on identifying transport paths in low porosity rocks based on the hypothesis that grain boundary widening accelerates reactions in which one mineral is replaced by another (replacement reaction). We conducted a time series of replacement experiments of three limestones (CaCO_3_) of different microstructures and solid impurity contents using FeCl_2_. Reacted solids were analyzed using chemical imaging, small angle X-ray and neutron scattering and Raman spectroscopy. In high porosity limestones replacement is reaction controlled and complete replacement was observed within 2 days. In low porosity limestones that contain 1–2% dolomite impurities and are dominated by grain boundaries, a reaction rim was observed whose width did not change with reaction time. Siderite (FeCO_3_) nucleation was observed in all parts of the rock cores indicating the percolation of the solution throughout the complete core. Dolomite impurities were identified to act as nucleation sites leading to growth of crystals that exert force on the CaCO_3_ grains. Widening of grain boundaries beyond what is expected based on dissolution and thermal grain expansion was observed in the low porosity marble containing dolomite impurities. This leads to a self-perpetuating cycle of grain boundary widening and reaction acceleration instead of reaction front propagation.

## Introduction

The replacement of one mineral by another via dissolution-reprecipitation often occurs when minerals are in contact with surrounding solutions^1–3^. A fundamental understanding of this mechanism is important for a range of problems, including fractionation of rare earth elements, sequestration of radionuclides or heavy metals, remediation of legacy waste and formation of mineral deposits by reaction with CO_2_-rich mineralizing fluids. Previous research indicated that the microstructure of a monomineralic rock can influence its replacement rate^4–6^. In high-porosity rocks, replacement is accelerated due to the large surface area provided by porosity. In low-porosity rocks, other microstructural features, such as grain boundaries^7^ and twin boundaries^8^ become more important as they control reactive surface area. Weber et al.^5^ showed that grain boundary networks provide a fast pathway for fluid transport even at intermediate subsurface temperatures (200 °C), with rates intermediate between solid state diffusion rates and liquid self-diffusion.

Prior studies of natural systems have shown that replacement of calcite by siderite proceeds via transport along twin boundaries^8^. Many studies have investigated various dissolution/(re-)precipitation reactions (see reviews by Ruiz-Agudo et al.^1^; Altree-Williams et al.^2^), however the influence of solid impurities on these reactions has previously not been studied. In growth and dissolution experiments, both impurities present in the calcite and in the solution have been demonstrated to change calcite dissolution and growth/precipitation rates^9–12^. It can therefore be expected that the presence of solid impurities will alter the replacement reaction rate. Here, we used the model system CaCO_3_-FeCO_3_ to study the influence of solid impurities (in this case secondary phases with a volume concentration of 1–2%) on siderite (FeCO_3_) formation. At 200 °C the solubility of siderite (K_FeCO3, 200C_ = 10^–13.72^)^13^ is two orders of magnitude less than that of calcite (K_CaCO3, 200C_ = 10^–11.29^). These have the same structure (space group $R\overline{3 }c$), there is a wide solvus between them, and the Fe end-member of the ordered-intermediate dolomite-ankerite series (space group $R\overline{3 }$) does not exist^14^.

To investigate the effects of microstructure on ferrous iron transport and siderite precipitation in limestone we performed replacement/exchange experiments on three limestones/marbles with varied microstructures: a high-purity low porosity limestone (Carthage Marble, CM), a high porosity limestone (Texas Cream, TC) and a low porosity marble (Carrara marble, CAR) containing 1–2% dolomite to compare the effect of microstructure and solid impurities on replacement. Cores of these materials were subjected to time-series replacement experiments and the reaction products and textures were analysed to quantify porosity changes using inverse-space scattering combined with quantification of grain boundary widening rates via real-space electron microscopy imaging. Newly formed phases were identified via wide angle X-ray scattering (WAXS), SEM–EDS and Raman spectroscopy. Both spatially resolved X-ray and neutron small- and ultra-small angle scattering techniques ((U)SAXS and (U)SANS) were employed to quantify changes in pore sizes structures 10 nm to 2 μm for (U)SAXS and 10 nm to 20 μm using (U)SANS. We hypothesize that in a pure limestone, the reaction proceeds via a simple dissolution-reprecipitation reaction, whereas the presence of secondary impurity phases in the limestone will change the reaction process by providing alternate nucleation sites.

## Results

### Observation of the replacement of calcite by siderite using SEM

**Figure 1:**
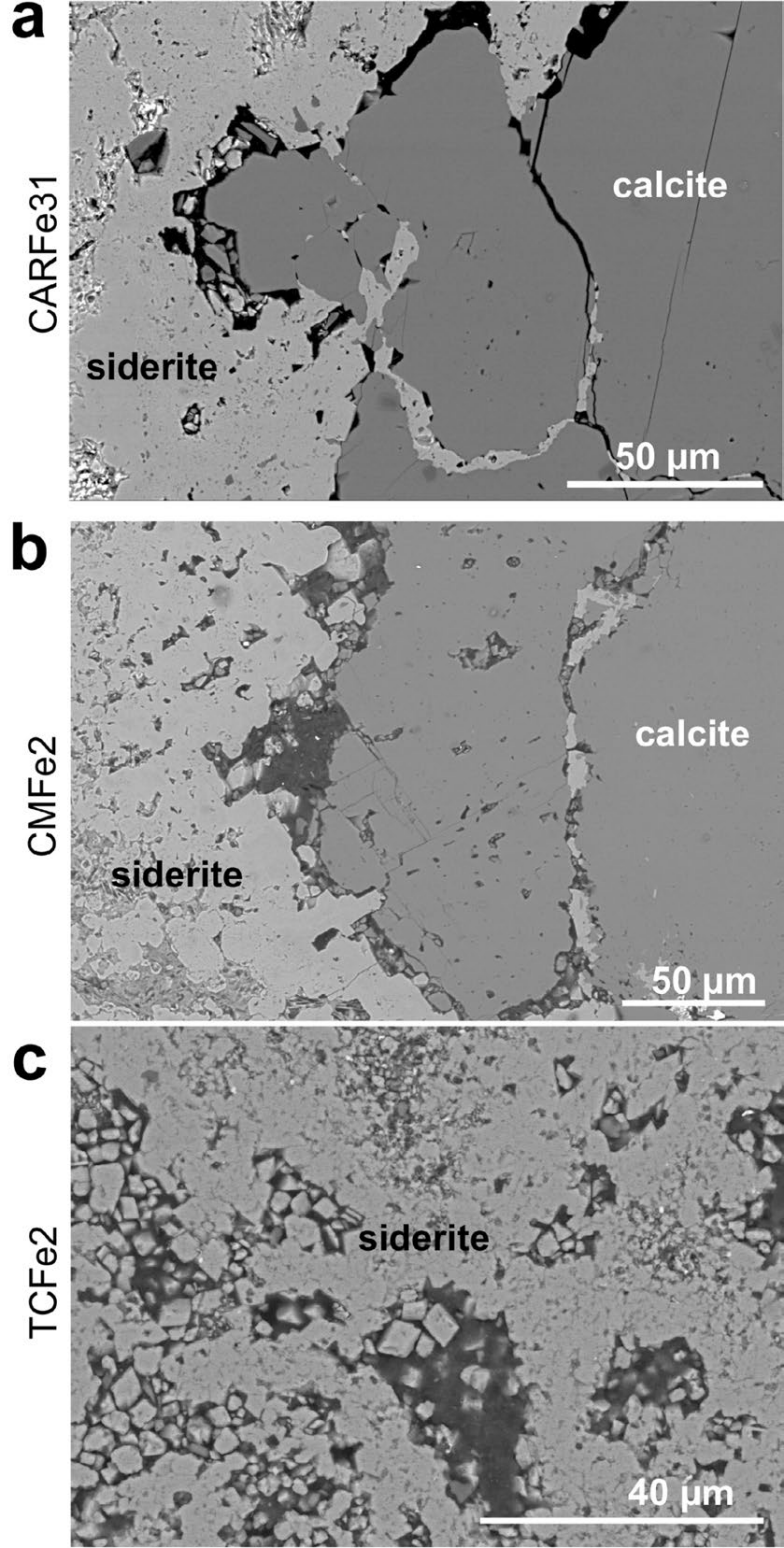
Backscattered electron (BSE) contrast images of the reaction interface between the original calcite and the replacing phase, siderite. (**a**) CAR marble reacted for 32 days, and (**b**) CM limestone reacted for 2 days. (**c**) shows the middle of the TC sample reacted for 2 days. Only siderite is visible which indicates complete replacement.

Depending on the microstructure of the starting material, we observed partial or full replacement of the original limestone by siderite. For the high porosity TC limestone reaction time for complete replacement is 2 days. After reaction of the solid with saturated solution, a reaction rim had formed consisting of siderite. Phase identification of siderite in rim was performed using Raman spectroscopy (see supporting information). SEM images (Fig. 1) did not show any other phases formed at the interface between original limestone and newly formed siderite. At the outside of the reacted core, most samples showed an additional rim of ferrihydrite (identified in TEM images, see supporting information) likely caused by exposure of reacted cores to oxygenated, deionized water after reaction. As this ferrihydrite rim is due to treatment after the experiment and not part of the replacement reaction, it will not be further discussed.

The overall reaction for replacement in this experiment is:

1 $${\text{CaCO}}_{{3}} + {\text{FeCl}}_{{2}} \to {\text{FeCO}}_{{3}} + {\text{ CaCl}}_{{2}}$$

**Figure 2:**
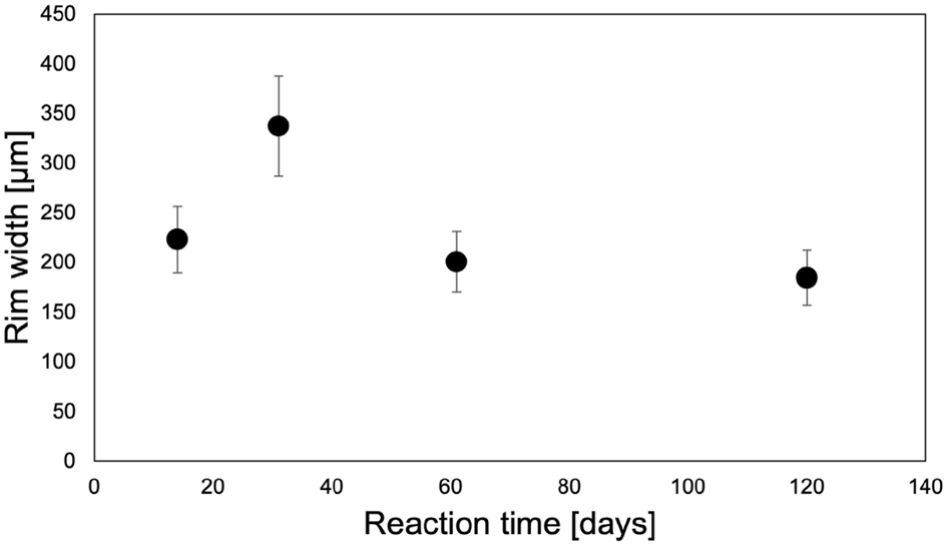
Reaction rim width as a function of time for CAR measured in SEM images.

During this reaction, the molar volume of the solid is reduced by ~ 20.5% from 36.934 (calcite^15^) to 29.378 cm^3^/mol (siderite^15^). Significant textural differences are observed in the replacement reactions for the three carbonate rocks (Fig. 1). For the Carrara Marble, in which the reaction is expected to be grain-boundary transport dominated because of its overall low porosity and permeability, the reacted rock cores showed a reacted rim composed of porous FeCO_3_. Between the original calcite and the replaced material, a gap of several μm was visible. No transport along twin boundaries was observed in either SEM images or EDS mappings, although twin boundaries were observed in polarized light microscopy as expected (see supporting information). The total reaction rim width was measured in SEM (Fig. 2) and was ~ 200 μm with no significant change with longer reaction times.

**Figure 3:**
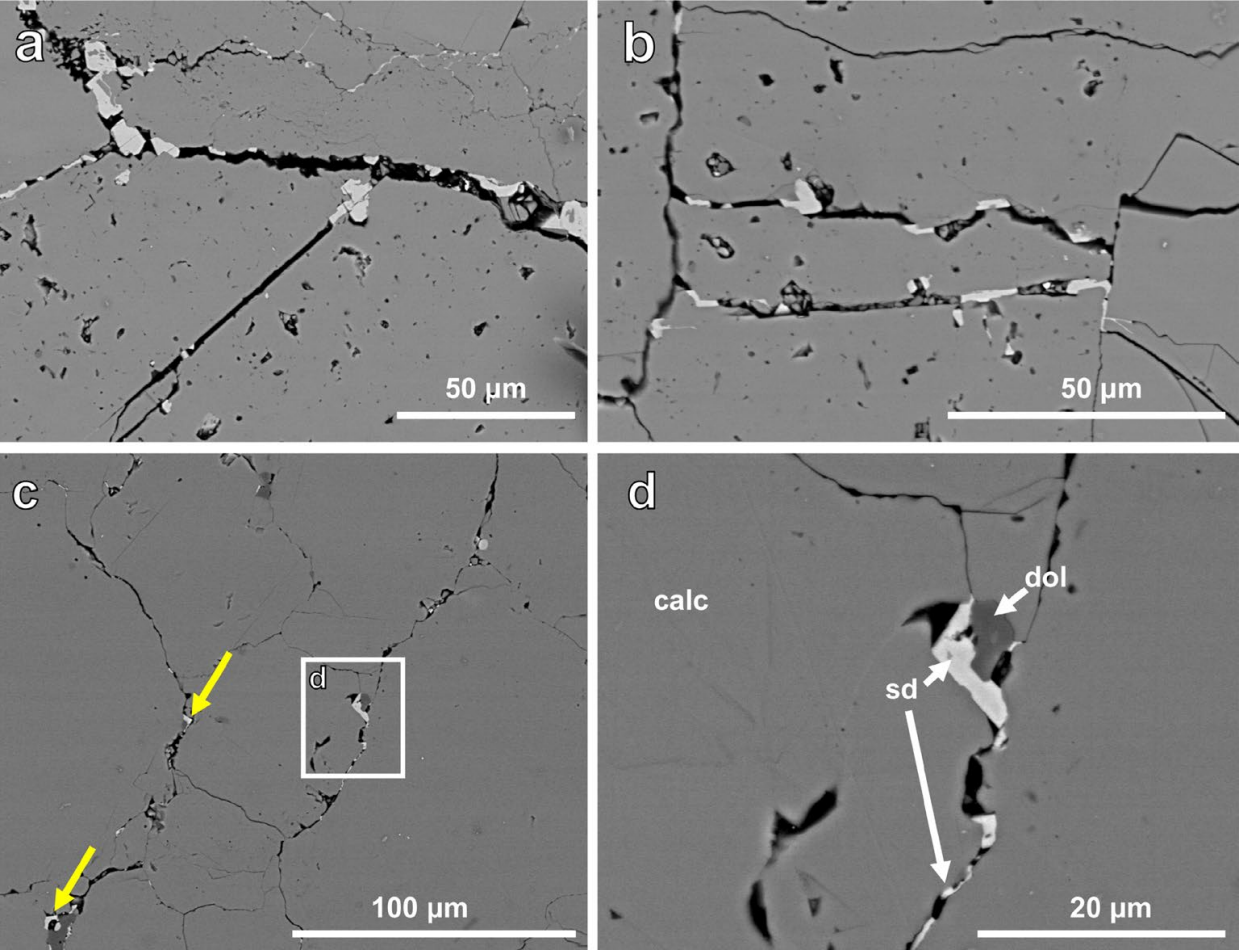
SEM-BSE images of CM and CAR showing. (**a**) straight cracks which are unlikely pre-existing and coincidence with siderite growth, (**b**) perpendicular cracks. (**c**,**d**) siderite nucleation on dolomite.

The reacted CM limestone showed a siderite reaction rim, separated from the original calcite by pores of 30–40 μm, similar to CAR limestone (Fig. 1). Siderite was observed to be preferentially located along pre-existing porosity or boundaries between grains (Fig. 3).

In the Texas Cream limestone, complete replacement by siderite was observed within 2 days. No reaction rim was visible. In addition to the overall reaction rims described above, all reacted cores of the Carrara Marble (14 days to 120 days reaction) contained small precipitates (100 nm to several μm) of siderite (identified using Raman spectroscopy and EDS available in supplementary information) in the interior of the core, often located next to the dolomite impurities (Fig. 2).

### Weight changes during control experiments

**Figure 4:**
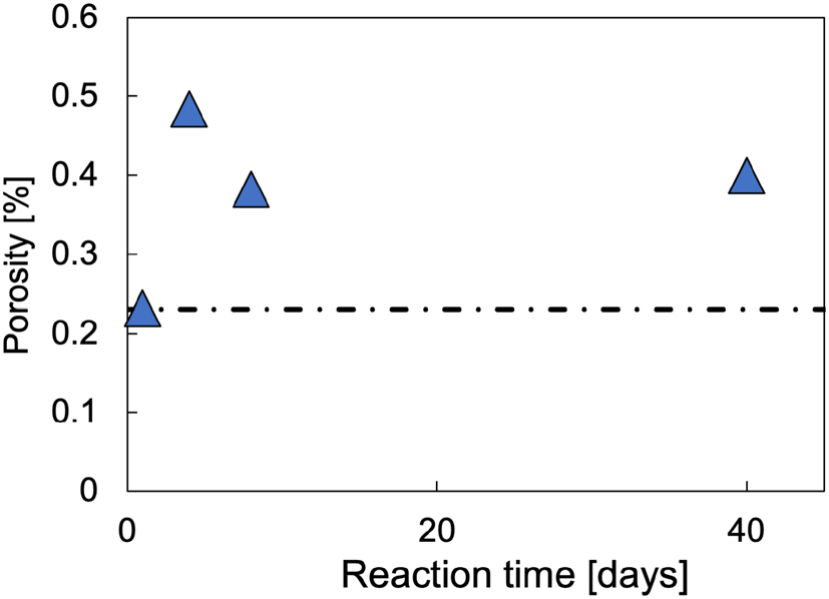
Porosity changes based on weight change during CAR dissolution experiments. Error bars are smaller than symbols. Streaked line indicated pristine CAR marble porosity.

The weight changes during the CAR marble control experiments were recorded and based on assuming calcite density of 2.71 g/cm^3^ converted into volume-based porosity changes. Resulting porosity (Fig. 4) increased to 0.5% within 4 days but at 8 days porosity was 0.4%. When calculating how much calcite would dissolve in the amount of liquid available (~ 5 ml, with minor changes depending on amount of salt added), the amount calculated (0.00212 g) based on a logK_sp_ = − 11.29 of calcite at 200 °C^16^ closely matches the measured weight changes (1 day—0.0026 g, 4 days—0.0045 g, 8 days—0.0025 g and 40 days—0.0028 g).

### Quantifying phase evolution by wide angle X-ray spectroscopy

WAXS spectra show characteristic peaks for different minerals, similar like (powder) X-ray diffraction and recorded spectra were matched against mineral spectra available in the RRUFF^17^ database. The WAXS spectra of pristine CAR limestone showed characteristic peaks for calcite and dolomite (see supplementary information). No siderite peaks were detected in the reacted CAR cores, despite the observation of siderite nucleation within the limestone core in SEM images, indicating that the amount of siderite present was small. However, siderite was clearly identified using Raman spectroscopy as the bright reaction rim visible in SEM-BSE imaging (Fig. 1). In the CM limestone, a reaction rim is visible in the SEM images as well as in the WAXS spectra. In the 2-day experiment siderite diffraction peaks were observed in the outer ~ 2 mm, whereas in the rest of the sample, only calcite was observed. Complete replacement by iron-bearing phases was observed within 2 days in the high-porosity Texas Cream limestone. WAXS analysis of this material showed that the newly formed phases were primarily siderite, although minor amounts of goethite were present. Samples reacted for 14 and 31 days show complete replacement by siderite in most of the rock. However, some ferrihydrite was present in the outer layer. To test whether ferrihydrite formation was due to overnight exposure to water during post-experimental washing three 2-day experiments were conducted and instead only washed 3 times for 1 min each with deionized water before drying. Ferrihydrite formation was not observed, suggesting that it might have formed during the longer rinsing stage, possible by reaction with fine-grained siderite as reported in literature^18^.

### Quantification of porosity changes by (U)SAXS and (U)SANS

**Figure 5:**
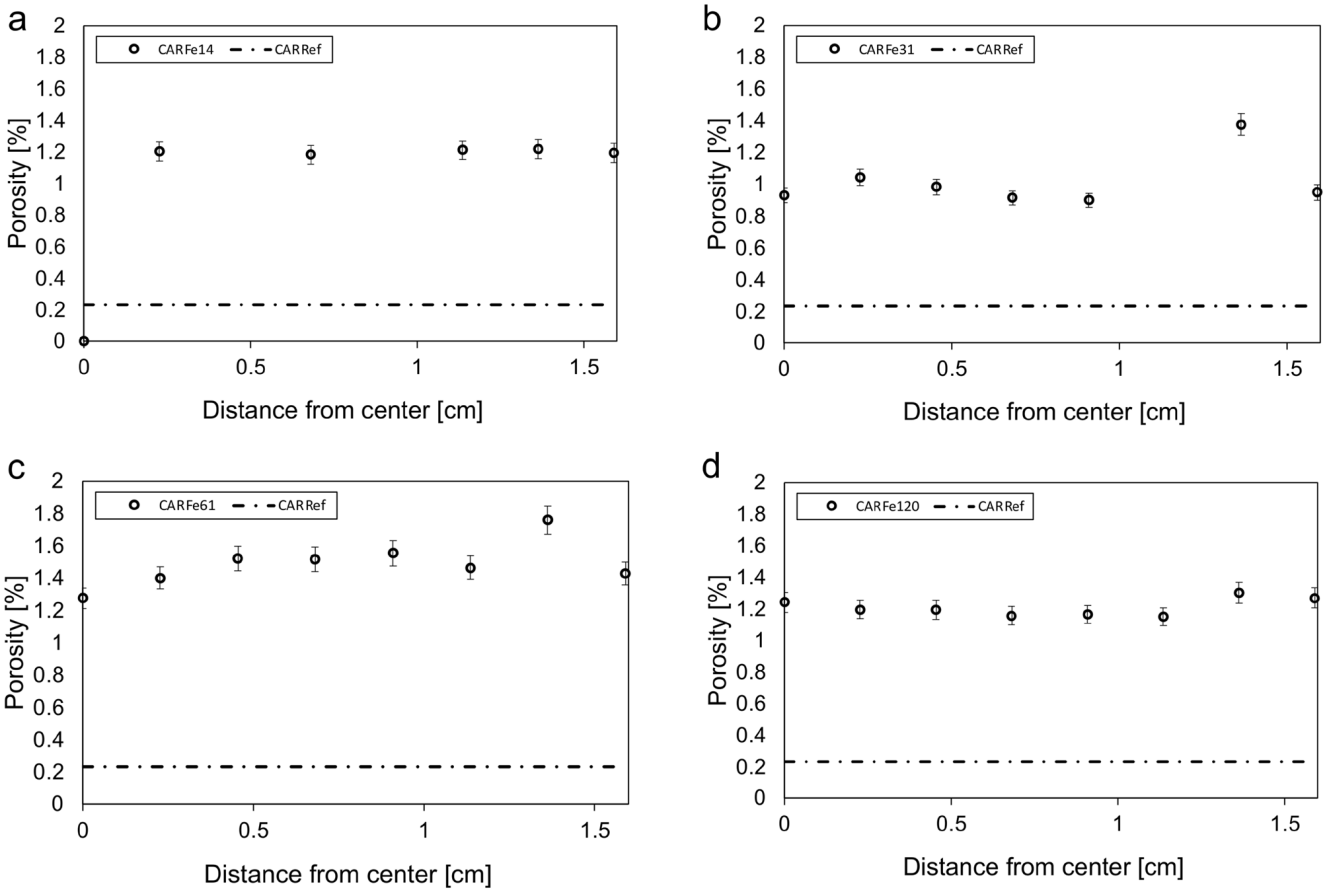
Porosity development in the CAR marble with reaction time as characterized by (U)SAXS. Porosity is elevated in comparison to the pristine sample, but no rim is visible.

**Figure 6:**
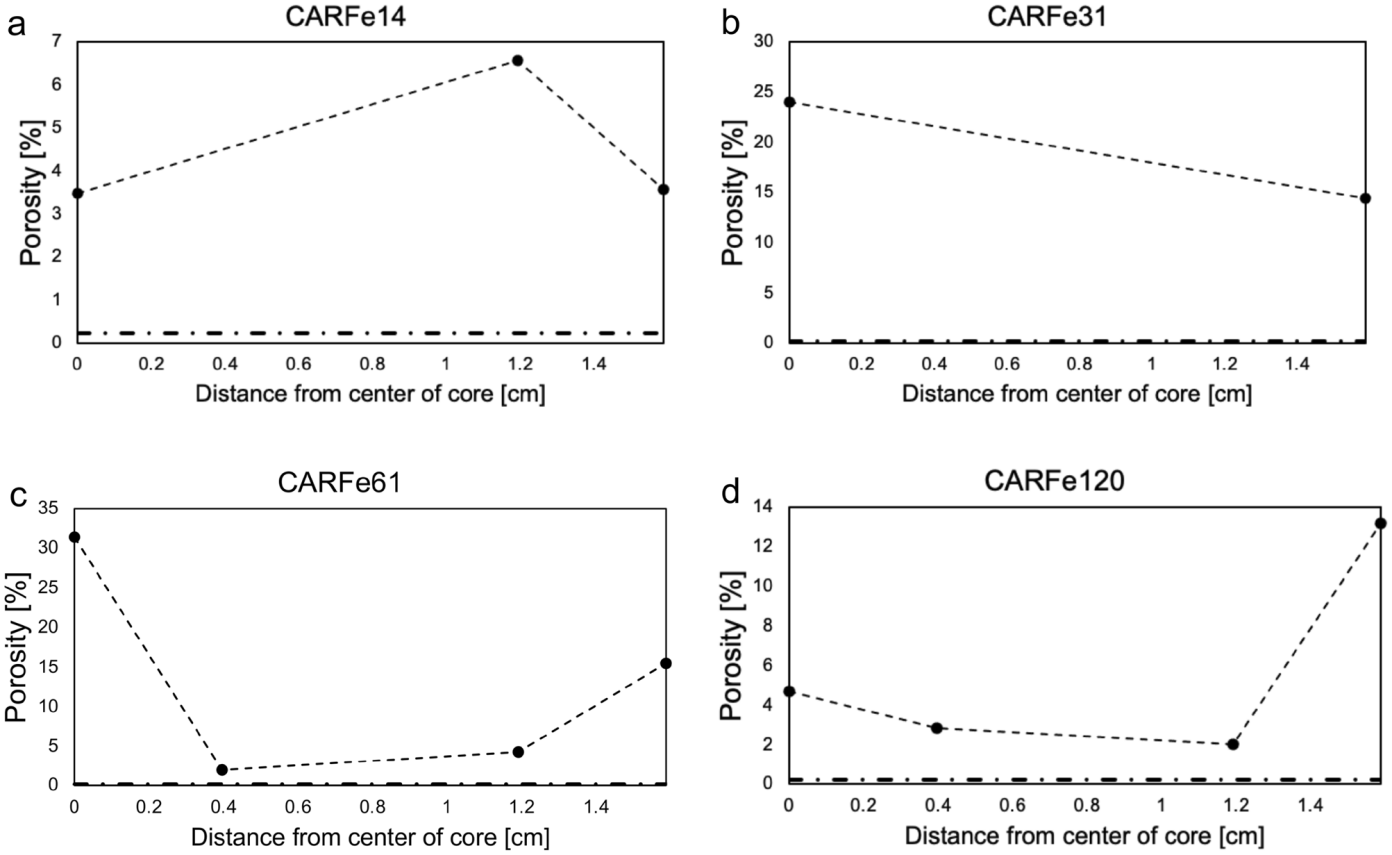
(U)SANS results for CAR. Porosity is plotted as a function of distance from center of the rock core for (**a**) CARFe14, (**b**) CARFe31, (**c**) CARFe61 and (**d**) CARFe120. Horizontal dashed-dotted line indicates porosity in pristine CAR marble samples.

There are significant differences in the change in porosity observed in the three carbonate rocks after reaction. In comparison to the pristine Carrara marble (porosity: 0.3% measured by (U)SAXS), the reacted samples showed an elevated porosity of ~ 1.2% ± 0.3 in (U)SAXS characterization (Fig. 5). The resulting porosity is approximately double the porosity determined by weight change for CAR dissolution experiments. This indicates that dissolution alone is not responsible for higher porosity. The larger values from (U)SANS are expected, as (U)SANS has a higher pore size detection limit (Fig. 6). Spatial analysis by both techniques suggests that this is relatively homogeneously distributed throughout the sample. No elevated porosity was detected at the rim and porosity did not increase with reaction time. As each reaction time involved exposure of a different sample, the latter suggests that this is not simply due to variability in the porosity of the starting material. In addition, as discussed above, the relative similarities of the results obtained for the starting material from (U)SAXS, water saturation, and previous work^19,20^ suggest that, at least initially, there are few large pores in this material outside the sensitivity range of (U)SAXS and (U)SANS, and there was no evidence of their formation in SEM imaging. However, as (U)SAXS detects all empty space within the rock, widening of grain boundaries will be classified as an increase in overall porosity.

**Figure 7:**
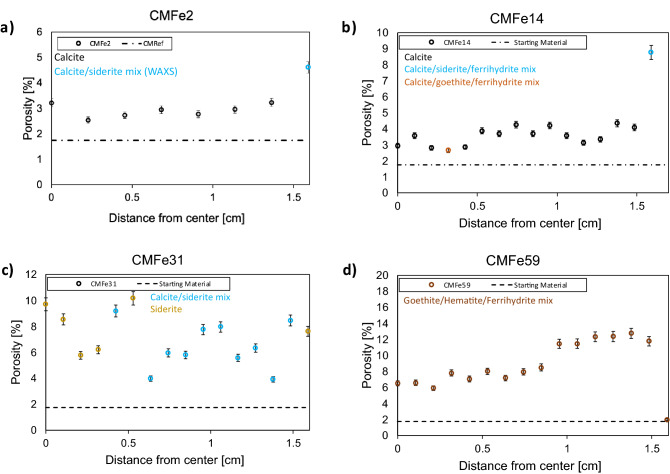
Porosity development in the CM limestone with reaction time as characterized by (U)SAXS. Phase determination as determined by WAXS is given in the color code.

In the CM limestone, porosity was elevated to ~ 2.5–3% throughout the core after 2 days of reaction, with a rim of ~ 5% porosity (Fig. 7). WAXS analysis showed the formation of siderite in the rim and in the SEM images (Fig. 1), newly formed pores in the siderite rim are visible. After 14 days reaction, the overall porosity was elevated to ~ 3–4% with a rim of ~ 9% porosity. In the WAXS analysis, siderite/ferrihydrite mix was observed in the rim, whereas the rest of the core was detected as calcite only. However, in SEM-BSE analysis the formation of siderite throughout the core was observed (Fig. 3). The samples of CM limestone reacted for 32 and 59 days were completely reacted, with primary phases present being siderite, goethite, hematite and ferrihydrite. Porosity was elevated to 4–10% after 32 days and between 6 and 12% after 59 days (Fig. 7). TC limestone did not show significant changes in porosity (see supporting information), which is likely due to its high starting porosity.

### Grain boundary width analysis in carrara marble by atomic force microscopy and scanning electron microscopy image analysis

**Figure 8:**
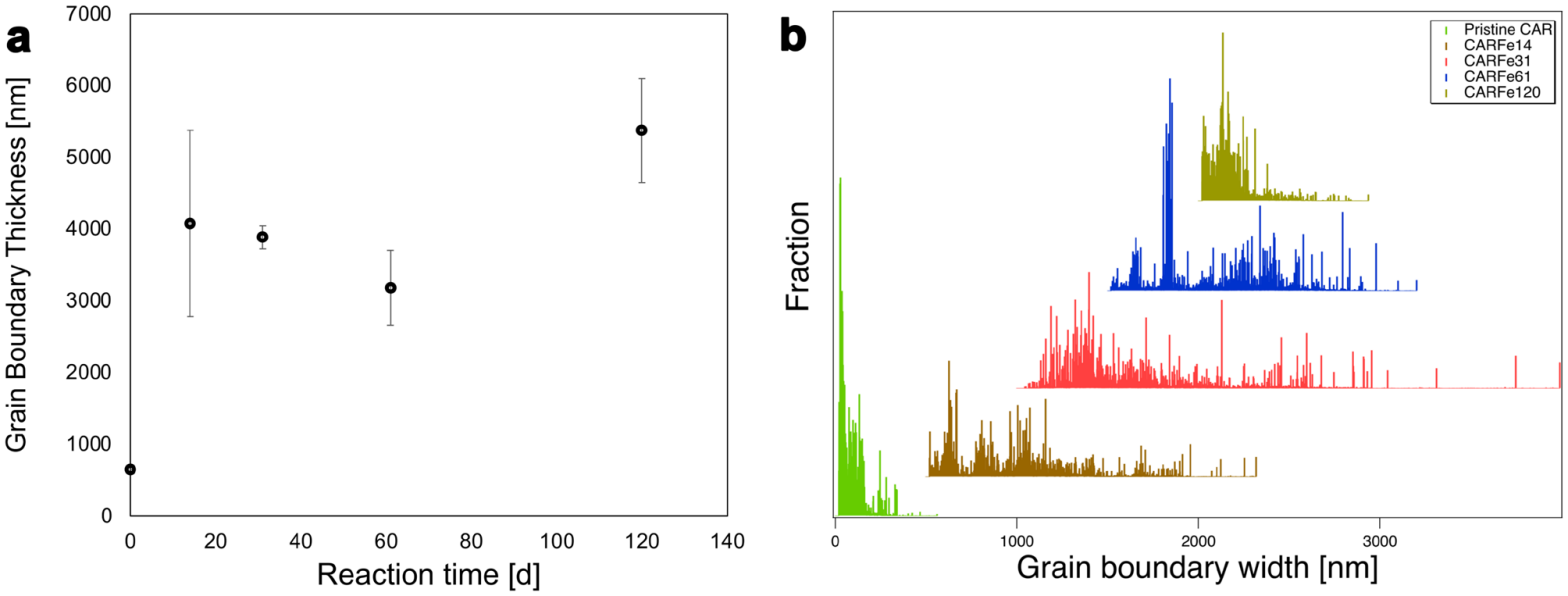
(**a**) Average grain boundary width in nm as a function of reaction time in days measured by AFM. (**b**) Grain boundary thickness distribution determined by SEM image analysis via local thickness analysis. CAR Reference before reaction is plotted in green, CAR reacted for 14 days in brown, CAR reacted for 31 days in red, CAR reacted for 61 days in blue and CAR reacted for 120 days in light green.

The average grain boundary width measured by AFM in the pristine Carrara Marble sample was 642 ± 163 nm (Fig. 8). The observed variability probably reflects both actual variations in grain boundary width and artificially larger values measured because the plane of the thin section is not always perpendicular to that of the grain boundary. After reaction with FeCl_2_, all samples show an elevated grain boundary width: 4073 ± 724 nm after 14 days of reaction, 3471 ± 519 nm after 32 days of reaction and 5368 ± 1297 nm after 120 days of reaction. Within error, these are larger than the value for the starting material, but otherwise similar. This is consistent with the (U)SAXS estimation of a single change in the pore volume (starting vs. reacted) and no change with time. Nonetheless, the alteration of the starting material is likely to influence fluid diffusion rates along the grain boundaries and, therefore, longer-term replacement.

Since AFM measurements tend to overestimate the grain boundary thickness because it cannot detect grain boundaries smaller than the tip diameter (~ 3–4 nm), grain boundary widths were additionally determined from SEM images using the local thickness function in ImageJ. The average grain boundary width for pristine Carrara marble determined in this manner was ~ 82 nm, significantly lower with the AFM. The average grain boundary width increased to ~ 496 nm after 14 days reaction time and to ~ 736 nm after 31 days. These are also significantly lower than values obtained by AFM analysis but, again, show a significant increase relative to the pristine sample. The Carrara marble sample reacted for 59 days had an average grain boundary width of ~ 592 nm. After 120 days, the average grain boundary width again decreased to ~ 169 nm. However, the measured values were not normally distributed, and the spread of the data needs to be considered. The maximum grain boundary width in the pristine CAR sample was ~ 561 nm, whereas those for the reacted samples were more elevated (CARFe14 = 1817 nm, CARFe31 = 2989 nm, CARFe61 = 1700 nm, CARFe120 =  ~ 935 nm). The grain boundary size distribution is plotted in Fig. 8b and shows that for both samples reacted for 31 and 59 days, the distribution is significantly broadened. The asymmetry towards high values is the effect expected due to non-perpendicularity.

## Discussion

Both the low porosity Carthage marble limestone and high porosity Texas Cream limestone reacted fast, i.e., complete for TC within 2 days of reaction and CM samples within 14 days. These rates are much faster than observed for Mg or F replacement of the same materials^4,5^. No trend in reaction rates is observed based on solubility differences or molar volume change between endmembers (see supporting information for graphs).

One possible reason for differences in replacement speed between these three different systems is the difference in endmember crystallography. Siderite and calcite are both in the same space group, R$\overline{3 }$c, and differences between calcite (a = 4.898 Å, c = 17.062 Å) and siderite (a = 4.72 Å, c = 15.72 Å) unit cell parameters are smaller than differences between calcite and dolomite (space group R$\overline{3 })$ or fluorite (space group F m3m). It can therefore be concluded that the degree of similarity in crystallographic structure between the parent and product system is also important for the replacement speed since it promotes epitaxial nucleation^21^.

One of the differences observed between the three starting materials was the mineralogy of the replacement phases. In the Carthage Marble a direct replacement of calcite by siderite was observed. In the 2-day experiment, only siderite and calcite were observed, whereas in the 14-day experiment a goethite/ferrihydrite mix was present. CMFe59 was completely reacted to a goethite/hematite and ferrihydrite mixture.

TC limestone was completely by siderite replaced within 2 days, with the 2-day and 14-day sample showing only siderite. The outermost rim showed the presence of ferrihydrite, here as well likely caused by post experiment treatment of the rock cores. TC limestone samples reacted for 59 and 120 days showed a mixture of hematite, siderite, and goethite (59 days) and a mixture of calcite, hematite, goethite, and magnesite (120 days). It is unclear why calcite remains present in the 120-day sample, one possibility is that mineral grains become armored by reacted phases and are inaccessible to the reactant^22^.

In addition, in the CAR marble experiments transport appeared to be dominated by the grain boundary structure. EDS characterization did not show any transport of Fe along twin boundaries as observed by Pearce et al.^8^ on a sample from the 1.95 Mya Junction gold deposit, Western Australia, in which calcite derived from carbonation of a metamorphic amphibole and plagioclase assemblage was altered to siderite and dolomite. This suggests that, in our experimental materials, either grain boundaries or other connected porosity provided a much more facile pathway for reaction and therefore the amount of Fe transported along twin boundaries might be below the detection limit. Another possibility is that the twin boundaries were more open in the replacement-derived samples analyzed by Pearce et al.^8^. In addition, Raman spectroscopy shows that there is likely a low percentage of Fe incorporated into calcite, which is consistent with the CaCO_3_-FeCO_3_ phase diagram at 200 °C^14^. EDS was not able to detect Fe incorporated into CAR, which indicates that its concentration is likely below several hundreds of ppm (approximated EDS detection limit). Siderite rims on dolomite grains were also observed in a previous study on glauconite carbonation during sedimentary diagenesis^23^.

In volume-reducing dissolution-reprecipitation reactions, porosity formation within the newly precipitated phase^2^ and/or at the interface between original phase and new phase^24^ is commonly observed. Replacement of calcite by siderite is volume-reducing and porosity formation is observed within the newly formed siderite (Fig. 1, left side of image) and at the interface (Fig. 2). However, unlike the situation observed during Mg replacement of limestones and, by implication of its total replacement in our experiments, in the Carthage marble, only a very narrow replacement rim (~ 200 µm, Fig. 2) is observable in the reacted Carrara marble. The width of this rim does not appear to increase with reaction time, and we do not observe elevated porosity in the rim using scattering methods. However, SEM imaging (Fig. 1) shows the presence of large-scale (several μm) pores at the calcite/siderite interface. Thus, part of this could be due to the size limitations on (U)SAXS and (U)SANS sensitivity. In addition, as noted above, grain boundaries were observed to widen, increasing porosity, throughout the rock soon after reaction began. These are often associated with the growth of individual siderite grains within the matrix, often associated with dolomite. We hypothesize that the formation of preferential pathways and nucleation of individual siderites prevents further replacement from the rim by allowing these internal grains to absorb all available iron, the transport of which may be limited by the thin replacement rind.

Based on SEM-BSE image analysis, we calculated the replacement rate for each rock type, similar to previous publications^4,5^. Here, we assumed for a slice of the core 1 cm thick with a radius r_core_ = 0.79 cm, the volume of the core, V_core_, is equal to 1.98 cm^3^. After calculating the volume of the reacted rim by subtracting the unreacted inner core from V_core_, the replacement rate was calculated by dividing the V_reacted_ by the reaction time in seconds. R_replacement_ was then divided by the volume of the core, yielding a normalized replacement rate. Only minimum rates were obtained for TC limestone since it was completely replaced within 2 days of reaction. An overview of replacement rates is given in Table 1.

**Table 1:** Replacement rate based on SEM-BSE image analysis. V_core_ = 1.98 cm^3^.

Sample name	Reaction rim width-average [cm]	r_unreacted core_ [cm]	V_unreacted core_ [cm^3^]	V_reacted rim_ [cm^3^]	t_reaction_ [days]	t_reaction_ [s]	R_replacement_ [cm^3^/s]	R_norm_ [s^−1^]
CMFe2	0.01	0.78	1.92	0.06	2	172,800	3.2E−07	1.6E−07
CARFe14	0.02	0.77	1.87	0.11	14	1,209,600	9.1E−08	4.6E−08
CARFe31	0.03	0.76	1.81	0.16	31	2,678,400	6.1E−08	3.1E−08
CARFe61	0.02	0.77	1.88	0.09	61	5,270,400	1.9E−08	9.5E−09
CARFe120	0.02	0.78	1.89	0.09	120	10,368,000	8.8E−09	4.4E−09
TCFe2	0.79	Not applicable, completely reacted		1.98	2	172,800	1.1E−05	5.8E−06

SEM/AFM characterization of grain boundary widths in the reacted Carrara Marble samples suggests that replacement of low-porosity dolomite-bearing limestone by siderite is controlled by transport along grain boundaries, which is enhanced by siderite growth within grain boundaries that widens them. This results in a chemomechanical feedback loop that allows more subsequent siderite precipitation because of the enhanced transport within the widened grain boundary. While a siderite rim formed at the edge of the core, it was thin and did not grow consistently inward. Instead, after the initial rim formation, nucleation of siderite crystals in the middle of the rock core, coupled with disperse grain-boundary widening is observed. The observed grain boundary widening is detected in (U)SAXS and (U)SANS as higher porosities. The increase in porosity during replacement by siderite is significantly higher than during limestone dissolution alone. This can be explained by preferential nucleation of siderite onto the dolomite grains within the marble. The lattice mismatch between siderite and dolomite is ~ 21% lower than the lattice mismatch between calcite and siderite, therefore it is likely energetically favorable for siderite to nucleate on dolomite over calcite. The half-cell c-axis lengths for Mg-dolomite (16.01 Å, Graf^25^) and for ankerite (16.186 Å, 70% CaFe(CO_3_)_2_, Ross and Reeder^26^) are more similar to that of siderite (15.373 Å, Graf^25^) than is calcite (17.061 Å, Graf^25^). Additionally, the molar volume change between dolomite (V_Dol, 1CO3_ = 32.17 cm^3^/mol, Robie et al.^15^; V_Ank, 1CO3, est_ = 32.86 cm^3^/mol, Anovitz and Essene^14^) and siderite (V = 29.378 cm^3^/mol, Robie et al. 1978) is − 8.7% relative to Mg-dolomite (on a 1 CO_3_ formula basis) and − 10.6% relative to an estimated value for Fe-dolomite (ankerite, estimated because the end-member is not stable, Anovitz and Essene^14^). Either is significantly smaller than the volume change between calcite and siderite. This suggests that it is energetically more favorable for siderite to nucleate on dolomite than on calcite.

We observe nucleated siderite in the middle of the CAR rock core which indicates that the fluid has percolated completely through the rock within the timeframe of the shortest experiment (14 days). Using the radius of the experimental cores as a limit, this yields a minimum diffusion rate of 5.2 × 10^–11^ m^2^/s. By comparison, solid diffusion rates for calcium or magnesium in calcite can be estimated at no more than roughly D ~ 10^–30^ m^2^/s based on extrapolation of available literature data^27,28^. For the longest experiment duration of 120 days, this would be equal to a diffusion depth of 0.32 μm. Thus, solid state diffusion likely played little role in the iron transport. While the fact that there are no data available for Fe^2+^ diffusion in calcite might introduce minor inaccuracies, it is clear that the fluid has to have percolated along the grain boundaries, which provide a faster diffusion pathway than through the grains^29,30^. An experimental study by Etschmann et al. indicated the importance of grain boundaries in speeding up replacement reactions^31^. This is consistent with the results of Weber et al.^5^ on replacement of limestone by dolomite, who observed that grain boundary diffusion rate was ~ 10 orders of magnitude faster than solid state diffusion of the same element in the same material, very similar to the results obtained above. In addition, previous modeling studies have shown that dissolution in limestones occurs preferentially along grain boundaries^5,32^.

Both the AFM and SEM analyses suggest that grain boundary width distribution (difference between minimum and maximum value) increases with reaction time. This appears to be a chemomechanical effect due to the growth of siderite crystals along these boundaries. The forces exerted by the growing siderite crystals appear to be separating the calcite grains, widening grain boundaries, and forming preferential pathways for fluid transport. This may also be related to the fact that the width of the reaction rim in the Carrara Marble samples does not appear to increase with reaction time. This contrasts to both the results observed for the CM and TC samples, as well as previous results for Mg and F replacement^4,5^. Instead, a reaction rim with a nearly constant thickness around 200 μm was formed. This is likely due to differences in starting microstructure which influence transport and precipitation of siderite within the limestone core. Because Carrara Marble has been recrystallized, where Carthage “marble” and Texas Cream limestone have not, the very low permeability of the rock leads to slow dissolution and transport through both the original rock. This suggests that, after the initial alteration of the exposed edge of the core, the saturation index needed to overcome the energy barrier for heterogeneous nucleation of siderite can only be reached along certain wider pathways and relatively little iron will be transported even there. The lower energy barrier for nucleation on dolomite relative to calcite due to lower lattice strain will lead to a greater likelihood of siderite nucleation on dolomite grains, the growth of which will further increase the favorability of the transport path in question.

Comparing the average and maximum grain boundary widening rates with calculated siderite growth rates for different supersaturations (Jiang and Tosca^33^, details in supporting information) at ambient conditions (where siderite growth should be slower than at 200 °C), the free siderite growth rate is slightly higher than the grain boundary widening rate. This could be due to a dependence of the siderite growth on calcite dissolution and on the confining pressure exerted on the grains by the surrounding matrix, but the uncertainties in both the grain boundary and siderite growth calculations are also large enough to potentially account for the difference.

There must exist a chemomechanical equilibrium for siderite growing in fractures and grain boundaries in carbonate rocks. The growing siderite grain has to overcome the stress from calcite grains, but also exerts force on the surrounding crystals. In addition, both materials have a certain, orientationally-dependent, compressibility, leading to a degree of (visco-) elastic, and possibly visco-plastic behavior. Fracturing has previously been observed in rocks during replacement reactions (e.g., leucite by analcime^34^, pyrite by chalcopyrite^35^ as well as during MgO/CaO hydration reactions^36^), and fracturing by growth in grain boundaries is a well-known phenomenon (e.g., salt crystal growth in cements, seashore rocks and cements^37^). There are different ways to estimate the force exerted by the growing siderite: (1) the volume change approach and (2) the spring constant approach. The first assumes that an increase in molar volume leads to reaction-induced fracturing. This is suitable for many replacement reactions but, since siderite has a smaller molar volume than calcite, no force can be exerted based solely on a change in molar volume during replacement. Instead, the force exerted by the growing siderite crystal can be estimated based on a simple spring constant calculation via Hooke’s law. In the simple case this involves several assumptions/limitations: (1) the crystal started growing at the beginning of the experiment and continued to grow until the end, (2) the crystal has the shape of a sphere, (3) the force is unidirectional and does not spread in a ring-pattern and (4) the load does not exceed the materials’ elastic limit. The force exerted *F* over a cross-sectional area *S (F/S in N/m*^*2*^*)*can be estimated using:

$$\frac{F}{S}=Y\frac{\Delta l}{l}$$

With Y being the Young’s modulus of calcite (N/m^2^) and $\Delta$*l* the half of the grain boundary width, *l* being the starting grain boundary width. The compressibility of siderite is considered negligible. The Youngs modulus of calcite was previously obtained both experimentally^38,39^ and via simulations^40–42^. Reported values vary between 95 and 110 × 10^10^ N/m^2^. Using an average of 102.5 × 10^10^ N/m^2^ for the Young’s modulus of calcite, the exerted force was estimated based on crystal size in fractures/grain boundaries determined from SEM image analysis. For the CM limestone, this results in forces between ~ 1.5 × 10^5^ N/m^2^ (0.15 MPa) and 8.4 × 10^6^ N/m^2^ (8.4 MPa). When estimating a contact area of 10 μm^2^, the resulting force is between 1.4 10^–6^ N and 8.41 10^–5^ N. For CAR marble, based on the average grain boundary widths, the resulting forces are 1.7 × 10^5^ N/m^2^ (0.17 MPa) and 7.6 × 10^5 ^N/m^2^ (7.5 MPa). When estimating a contact area of 10 μm^2^, the resulting force is between 1.7 × 10^–6^ N and 7.55 × 10^–6^ N. Bending tests on Carrara marble showed a tensile strength of 14.91 MPa^43^. In nominal stress tests, maximum nominal stress values were between 6–7 MPa. Based on this approximation, it is therefore likely that the force exerted by the growing siderite crystal can cause the widening of the grain boundaries.

## Conclusions and implications

This study indicates that volume-reducing replacement reactions can lead to grain boundary widening and, potentially, to fracturing of rocks. This is in contrast to conventional wisdom that a volume-increasing replacement reaction is necessary^34,44^. A better understanding of what chemical reactions cause rocks to fracture is essential to assess caprock integrity for subsurface storage applications. This is important for analysis of the safety of subsurface CO_2_ sequestration^45^, as well as for geological storage of hydrogen^46^, and nuclear waste^47^ and recovery of geothermal energy^48^. Furthermore, this study shows that the reactivity of a material depends significantly on its microstructure, in particular its grain boundaries, which act as conduits for reactive fluids. Whereas the precise rates of solute transport and the correlation of those rates with specific reaction rates are beyond the scope of this study, it is clear that grain boundary widening has a significant feedback effect on overall rock reactivity that is active even in the short term. This interplay between transport, secondary phase precipitation, grain boundary widening and reaction volume changes may also have similar effects in other multigranular materials such as steels and other metallic materials, ceramics and nuclear fuel.

## Methods

### Characterization of starting materials

**Figure 9:**
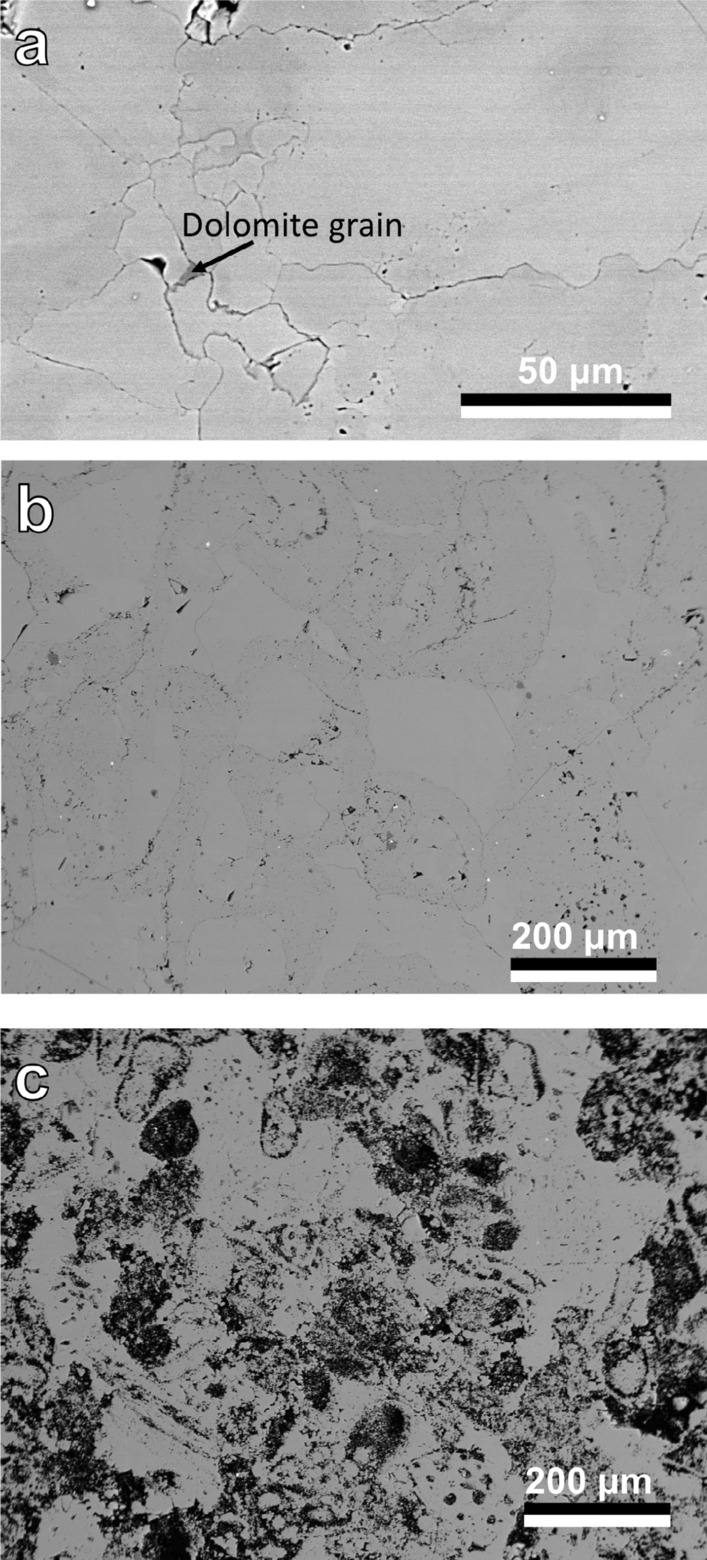
SEM-BSE image of (**a**) low porosity Carrara marble with highlighted dolomite grain, (**b**) low porosity limestone (Carthage marble) and (**c**) high porosity Texas Cream limestone. (**b**) and (c) reprinted from Weber et al.^5^, with permission from Elsevier.

Three limestones of different initial porosities were selected as starting materials for our experiments (Fig. 9). These are: (1) low porosity marble (Carrara marble, CAR), (2) low porosity limestone (Carthage marble, CM), and (3) high porosity limestone (Texas Cream, TC). Characterization of CM and TC has been reported in previous studies^4,5^ and the results are summarized here. Powder X-ray diffraction patterns modeled using Rietveld refinement showed that the low-porosity CM limestone was composed of ~ 100 wt% calcite, although traces of quartz were observed. The high-porosity TC limestone was composed of 99 wt% calcite, 0.5 wt% sylvite and 0.5 wt% quartz, with sylvite possibly stemming from bring inflow into the calcite. Carrara Marble, which was not described in the previous studies, was composed of 98% calcite and 2% dolomite. The latter was obtained from a sample of dimension stone known as “Carrara White.” A key difference between this material and the Carthage “marble” (also described as Burlington limestone) and the Texas Cream limestone is that it is a true marble. That is, the original limestone has been pervasively recrystallized metamorphically, leading to a material with larger, more consistent grains and a much tighter grain boundary network with a very low permeability^19,20^.

Initial porosity of the three carbonates was determined by both water saturation and (U)SANS. Water saturation measurements, in which the density of the initial sample and the same sample saturated with water by placing it under water in a vacuum are compared to that of pure calcite, suggested that the high-porosity TC limestone contained ~ 25 volume% porosity, whereas (U)SANS and (U)SAXS analysis yielded only ~ 8 volume% porosity. This discrepancy is most likely due to the presence of macropores in the TC limestone that are too large to be detected by (U)SANS. This is consistent with results from a study by Ghosh et al.^49^, who reported mercury injection capillary pressure measurements for the TC limestone indicating that a portion of the pores throats were in the 100–200 µm range, and total porosity and permeability values of 27.5% (reasonably consistent with our result) and 15 mD, respectively. Porosity determined by fluid saturation for the low-porosity CM limestone was 1.5%, in good agreement with the value of 1.8% determined by (U)SANS and 1.7% by (U)SAXS. The reported permeability of the CM limestone is 0.004 to 0.007 mD (Kocurek Industries). Porosity determined via fluid saturation for the Carrara marble was 0.7% and 0.3% determined by (U)SAXS. Previous studies report porosities of 2.7 ± 0.2%^50^ measured by He pycnometer, with 67 ± 4% of these pores smaller than 10 μm. Other studies report 1% open porosity^20^ and 0.5%^19^ measured via Hg intrusion porosimetry.

### Replacement and control experiments

An overview of the experiments conducted is given in Table 2. Both replacement experiments and dissolution experiments were conducted. In these experiments, cylindrical samples of all three carbonate rocks with diameters of 1.59 cm (5/8 in.) and lengths of 1.59 cm (5/8 in.) were reacted in steel pressure vessels with Teflon liners and a Teflon seal. In the replacement experiments, FeCl_2_ solution was prepared from high purity FeCl_2_⋅4H_2_O using Ar-sparged (for 2 h) deionized water with a concentration of 3.45 M (equal to a saturated solution of FeCl_2_). To keep the solution concentration constant and at saturation throughout the experiment at 200 °C regardless of reaction extent, additional FeCl_2_⋅4H_2_O salt was added to the bottom of the reaction vessels. In the dissolution experiments, the rock cores were reacted with deionized water at 200 °C. The Teflon^®^ liners were 1.9 cm (6/8 in.) in inner diameter and 4.9 cm (1 34/36 in.) tall, with a fluid volume of ~ 13.89 ml. The head space in the reaction vessel had a volume of ~ 5 ml and was filled with air. The vessels were sealed to avoid water loss during the reaction and preheated in boiling water at the start of the experiment to minimize the time needed for the vessel to reach the reaction temperature^51^. To identify which changes originate from the replacement reaction and which due to alteration by heating and dissolution, three control experiments were conducted, in which Carrara marble cores were reacted with deionized water.

**Table 2:** Overview of experiments conducted in this study.

Sample name	Rock type	Duration [days]
TC_Reference	TC	–
CM_Reference	CM	
CAR_Reference	CAR	
CMFe2	CM	2
TCFe2	TC	
TCFe14	TC	14
CMFe14	CM	14
CARFe17	CAR	17
TCFe32	TC	32
CMFe31	CM	31
CARFe31	CAR	31
TCFe59	TC	59
CMFe59	CM	59
CARFe61	CAR	61
TCFe120	TC	120
CARFe120	CAR	120
CARdiss_1	CAR	1
CARdiss_4	CAR	4
CARdiss_8	CAR	8
CARdiss_40	CAR	40

All replacement and control experiments were conducted at 200 ºC in a convection air furnace. Vessels were placed in a well in an aluminum block to assure thermal uniformity^51^. After removal from the furnace, each vessel was quenched in cold water and opened. The rock core was then rinsed, left in deionized water overnight to remove any remaining salt, and then dried in a vacuum oven. Weight differences between the initial and final state of each sample were recorded. Thin sections 150 µm thick mounted with epoxy on quartz glass were prepared from each sample by Spectrum Petrographics (see Anovitz et al.^52^). The sample for each section was taken across the center of the core (yielding a circular sample) to assure, to the extent possible, a nearly one-dimensional experiment.

Replacement of calcite by siderite requires that the iron remain ferrous despite the air in the headspace of the vessels. The qualitative redox state of the system was tested by reacting only FeCl_2_ solution in sealed vessels at 200 °C for 2 days. After 2 days, a color change from green to red was observed, indicating that at least some oxidation occurred. However, at room temperature and pressure (where the vessels were sealed), 5 ml of air contains only 4.4 × 10^–5^ mol of oxygen. The vessel contained ~ 6 ml of FeCl_2_ at 3.45 M, or approximately 0.0207 mol of ferrous iron. In addition, as noted above, extra ferrous iron chloride was added to maintain saturation during the reaction. Thus, the oxygen available in the air is far less than the ferrous iron added. The apparent color change can be attributed to the strong coloration caused by small amounts of ferric iron.

### SEM characterization

SEM characterization was performed using a Hitachi 4800 instrument at an accelerating voltage of 15–20 kV. Prior to analyses, samples were coated with a 10–15 nm thick carbon coating using a Cressington carbon coater. SEM image analysis for grain boundary width determination was performed using the local thickness plugin in ImageJ^53^ based on the analysis of the diameter of the largest possible inscribed sphere for the object and contains the point^54,55^. Prior to analysis, images were binarized and small clusters of pixels not part of the grain boundary were removed. A minimum of 10 SEM images per sample was analyzed. Details of the image analysis process are provided in the supporting information.

### SANS/USANS characterization

The details of the analytical approach used for our (U)SANS experiments have been reported in detail elsewhere^4,52,56–60^, and, therefore, are only summarized briefly here. The combination of SANS and USANS was used to probe porosity at scales ranging from 10 nm to 20 µm. SANS measurements were performed at ORNL’s High Flux Isotope Reaction (HFIR) CG-2 neutron scattering instrument^61^ and at the Spallation Neutron Source (SNS) USANS instrument BL-1A^62^. Additional SANS and USANS characterization was also performed at ANSTO’s neutron scattering instruments, Quokka (SANS, Wood et al.^63^) and Kookaburra (USANS, Rehm et al.^64^) respectively.

On CG-2 at HFIR, data at sample-to-detector distances of 1 and 7 m were measured with λ = 4.75 Å and data at 19 m were measured with λ = 12 Å. This latter extended the Q range to lower values, providing a better overlap with the USANS data at low Q. On Kookaburra, high resolution mode was used with λ = 2.37 Å. 29 mm diameter aperture size was selected for all measurement. Appropriate Cd mask background was subtracted from each data.

On Quokka, three detector configurations were used to achieve full Q-range of 0.0007 – 0.5 Å^-1^. For all samples, a 20 mm sample aperture was used. The following source to sample distances (SSD) and sample to detector distances (SDD) were used: (1) 20 m lens optics SSD with 20 m SDD using λ = 8.1 Å, (2) 12 m SSD with 12 m SDD using λ = 5 Å and (3) 12 m SSD with 1.3 m SDD using λ = 5 Å.

To spatially resolve the porosity using the (U)SANS instruments, we employed a set of annular Cd-masks as described in our previous papers (Weber et al.^4,5^; Anovitz et al.^65^, see setup shown in supplementary information file). SANS data were corrected for empty-beam scattering (with appropriate Cd mask), background counts, detector uniformity, sample transmission and scattering volume and reduced to an absolute scale relative to the intensity of the direct beam using python scripts.

SANS data were reduced by employing the NIST Center for Neutron Research SANS data reduction macros modified for the QUOKKA instrument, implemented in the IGOR software package^66^ and transformed to absolute scale by using an attenuated direct-beam transmission measurement^67^. For ANSTO, KOOKABURRA, data were reduced using in house software gumtree and then these slit-smeared data were desmeared using the Lake algorithm^68^. Data reduction and desmearing were performed using the Mantid data reduction software package^69,70^ for the HFIR and SNS data. SANS and USANS data were desmeared using the NIST Igor macros and merged to give full Q-range data. (U)SANS data was fit using the IRENA plugin in IgorPro^71^. Scattering length densities used to fit the (U)SANS data are given in the supplementary information file.

### (U)SAXS characterization

Small-, and ultra-small, and wide-angle X-ray scattering (SAXS, USAXS, WAXS) analysis was performed at the Advanced Photon Source at Argonne National Laboratory on beamline 9ID using 21 keV X-rays^72^. This is a combination of a Bonse-Hart USAXS instrument with SAXS and WAXS pinhole cameras. (U)SAXS provided data from ~ 1 nm to ~ 2 µm and WAXS extended this down to ~ 1 Å. Data were corrected for empty beam scattering and reduced using instrument-provided data correction routines for IGOR (NIKA, Ilavsky and Jemian^71^). USAXS data were desmeared to provide better continuity with the SAXS data during data analysis. The design of the instrument guarantees that the Q ranges of different segments overlap. Collected data were put on an absolute intensity scale to enable quantitative porosity characterization. For some samples, a rotating stage was utilized. Other samples were scanned with linescans across the middle of the sample to provide center-to rim analyses. X-ray scattering length densities utilized for (U)SAXS data fitting are given in the supplementary information. Data from both the neutron and X-ray scattering experiments were analyzed using the IRENA plugin^71^ for IGOR™.

### TEM characterization

TEM samples of the outermost reaction rim were prepared by scraping some of the reacted rim of the limestone sample, suspend them in ethanol and then dip-coat them onto lacey carbon grids. TEM images were collected using the high-angle annular dark field (HAADF) capability on a JEOL 2200FS microscope operated at 200 kV equipped with a high-angle annular dark field (HAADF) detector. Phase determination was performed via Fourier transformation of lattice-scale TEM images using DigitalMicrograph.

### Raman spectroscopy

Raman spectra were collected using a Renishaw InVia confocal Raman instrument equipped with a 532 nm laser, 1200 l/mm grating and edge filter. Peak positions were calibrated prior to data collection using a Si standard. Data analysis and visualization was done using Renishaw’s WiRE software. Phase identification was performed using CrystalSleuth^73^.

### Grain boundary width measurements via atomic force microscopy

The surface morphology of the unreacted CAR marble and four reacted samples (CARFe17, CARFe31, CARFe61 and CARFe120) were studied using atomic force microscopy (AFM). An Asylum Research MFP-3D instrument was used in contact mode with an PRP-TR-50 cantilever with a resonance frequency of 67 kHz and a force constant of 0.32 N/m. Since CAR marble is 98% composed of calcite, and dolomite grains exhibit a distinct, recognizable round shape different from calcite grains, the grain boundary width data obtained almost certainly stem solely from adjacent calcite grains and not from calcite/dolomite grain boundaries. In addition, prior to analysis limestone thin sections were studied under the light microscope to select analysis regions that were not part of the replacement rim. Grain boundaries appeared in the AFM surface morphology images as dark trenches and observed grain sizes were consistent with light microscopy and SEM observations. Depth profiles were obtained across the grain boundaries and grain boundary widths were computed from the depth profiles.

## Supplementary Information

Supplementary Information.

## Data Availability

The datasets generated during the current study are available from the corresponding authors on reasonable request.
