# Supplementary material for: Grain boundary widening controls siderite (FeCO3) replacement of limestone (CaCO3)
Source: Sci Rep. 2023 Mar 20;13:4581. doi: 10.1038/s41598-023-30757-y (PMC10027894; doi:10.1038/s41598-023-30757-y)
Supplement: Supplementary file 1 — Supplementary Information. [file 41598_2023_30757_MOESM1_ESM.pdf]

Supporting Information for
Grain Boundary Widening Controls Siderite ($FeCO_3$) Replacement of Limestone ($CaCO_3$)

Juliane Weber[1], Vitalii Starchenko[1], Jan Ilavsky[2], Lawrence F. Allard[3], Jitendra Mata[4], Lisa Debeer-Schmitt[5], Carolyn G. Cooke[6], Ken Littrell[5], Lilin He[5], Rui Zhang[1], Andrew G. Stack[1], Lawrence M. Anovitz[1]

[1]Chemical Sciences Division, MS 6110, Oak Ridge National Laboratory, Oak Ridge, TN 37830 USA, weberj@ornl.gov
[2]Argonne National Laboratory, Chicago, USA.
[3]Material Science and Technology Division, Oak Ridge National Laboratory, Oak Ridge, TN, 37820, USA.
[4]Australian Centre for Neutron Scattering (ACNS), Australian Nuclear Science and Technology Organisation (ANSTO), Lucas Heights, NSW, 2234, Australia.
[5]Oak Ridge National Laboratory, Oak Ridge, TN, 37830, USA.
[6]Tennessee Tech University, Cookeville, TN, 38505, USA.

Table of Contents

1 Optical Light Microscopy 3

2 (U)SANS Setup 4

3 Porosity Determination in CAR Dissolution Experiments Based on Weight Change 4

4 Wide Angle X-ray Scattering (WAXS) Spectra 5

5 Transmission Electron Microscopy Results 6

6 Additional (U)SAXS Results 7

7 Raman Spectroscopy Results 8

8 Grain Boundary Width SEM Image Analysis Details 8

9 Additional Information about Replacement Rates Differences 11

10 Comparison of Grain Boundary Width and Siderite Growth 12

11 References 13

1 Optical Light Microscopy

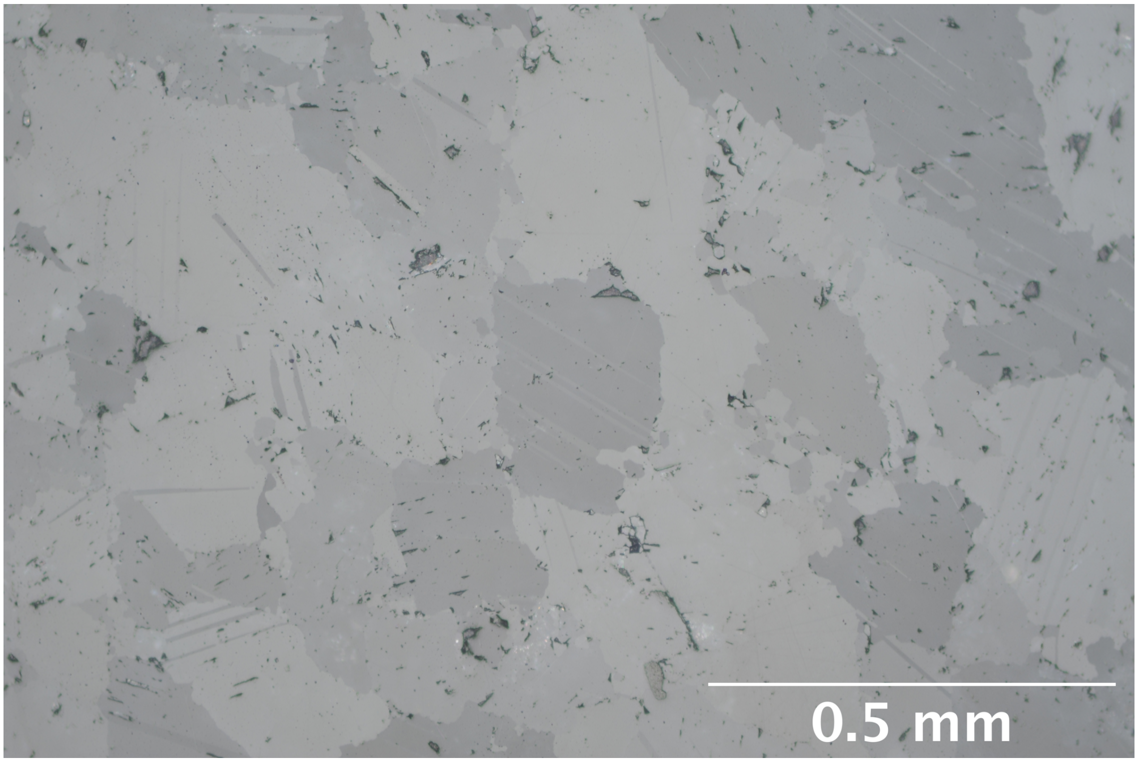

Fig. S 1 Polarized light microscopy image of Carrara marble thin section prior to experiments. Twin boundaries are visible in several grains.

2 Overview of X-ray and neutron scattering length densities

Table 1: X-ray and neutron scattering length density (SLD) for characterized materials.

Material	Calcite, Carthage Marble (CM)	Calcite, Texas Cream (TC)	Calcite, Carrara Marble (CAR)
X-ray SLD [10^{10}cm^{-2}]	22.97	22.93	22.98
Neutron SLD [10^{10}cm^{-2}]	4.72	4.71	4.73

3 (U)SANS Setup

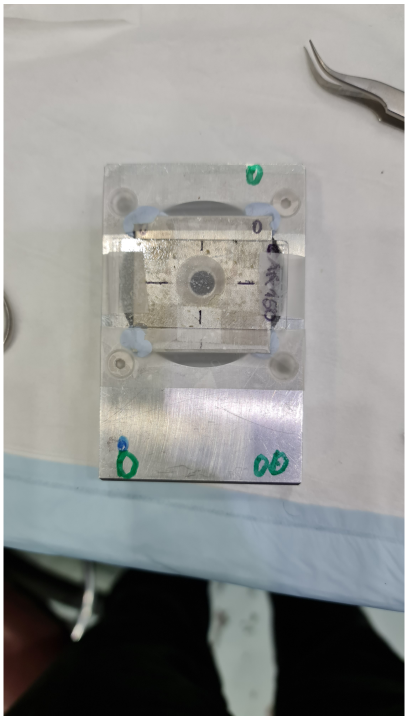

Fig. S 2 (U)SANS sample setup. Sample is attached to Cd-masks, which is attached to aperture.

4 Porosity Determination in CAR Dissolution Experiments Based on Weight Change

Experiment	duration (days)	Initial mass	Final mass	Change in weight [g]
CARdiss_1	1	6.9842	6.9816	0.0026
CARdiss_4	4	7.6035	7.599	0.0045
CARdiss_8	8	7.0375	7.035	0.0025
CARdiss_40	40	7.0858	7.083	0.0028

Weight change of Carrara marble cores were based on conversion of mass to volume based on density of calcite (2.71 g/cm^3). Volume of the rock core was calculated based on r = 0.79 cm and h=1.58 cm.

5 Wide Angle X-ray Scattering (WAXS) Spectra

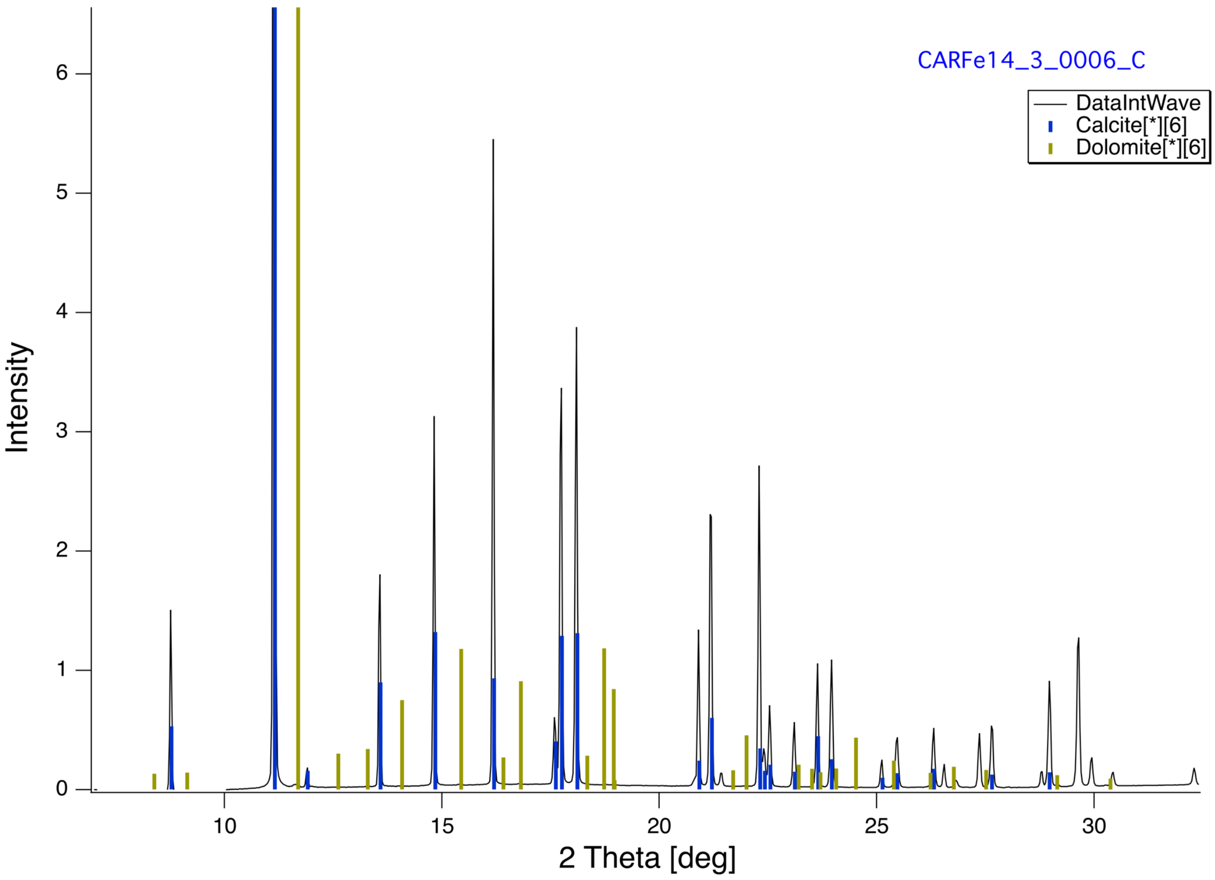

Fig. S 3 Representative WAXS spectra of CARFe14 showing characteristic dolomite and calcite peaks.

6 Transmission Electron Microscopy Results

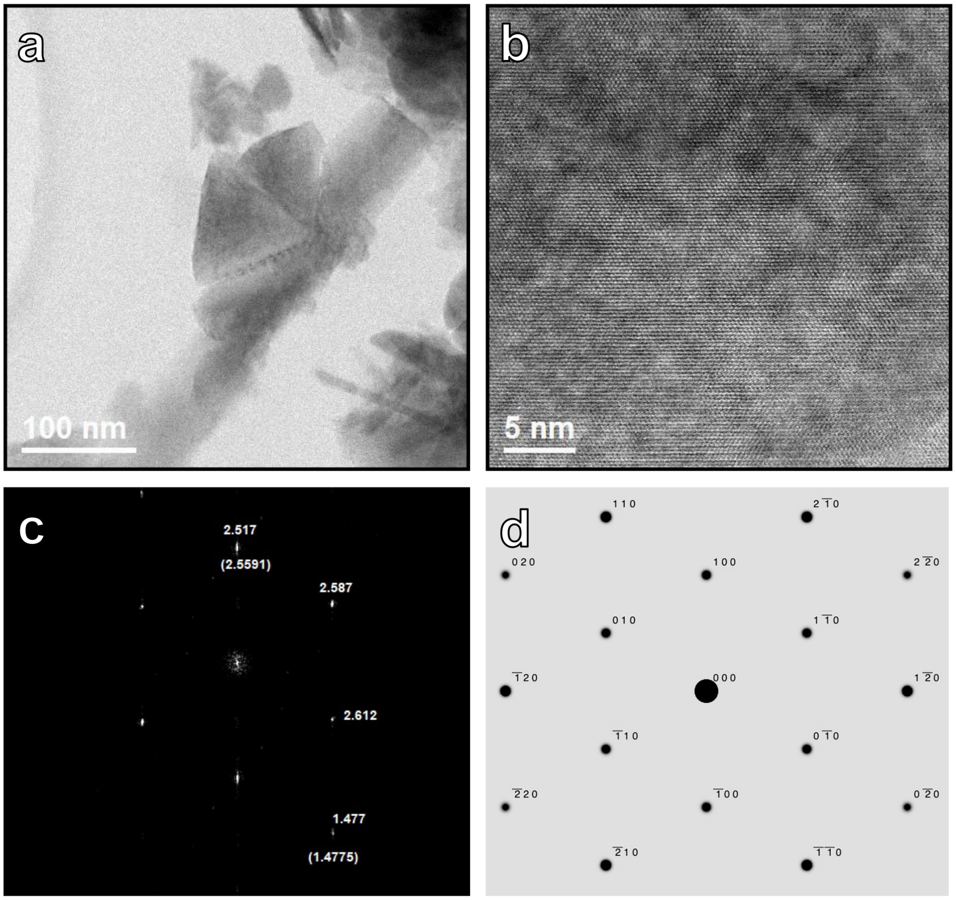

Fig. S 4 Scanning transmission electron microscopy results. (a) Bright-field (BF) STEM image of precipitated crystals. (b) BF STEM image of crystal lattice (c) Diffractogram of image in (b), consistent with the <0001> zone axis (c-axis) crystal orientation; actual d-spacings are shown in parenthesis. (d) Calculated diffraction pattern of ferrihydrite for the <0001> zone axis.

7 Additional (U)SAXS Results

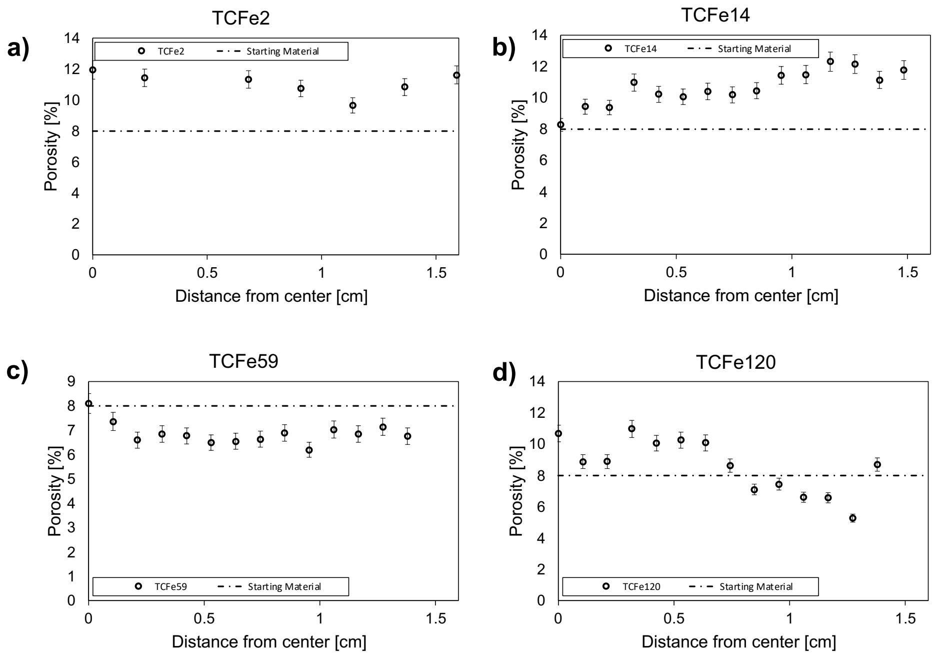

Fig. S 5 Porosity development in TC limestone with reaction time as characterized by (U)SAXS.

8 Raman Spectroscopy Results

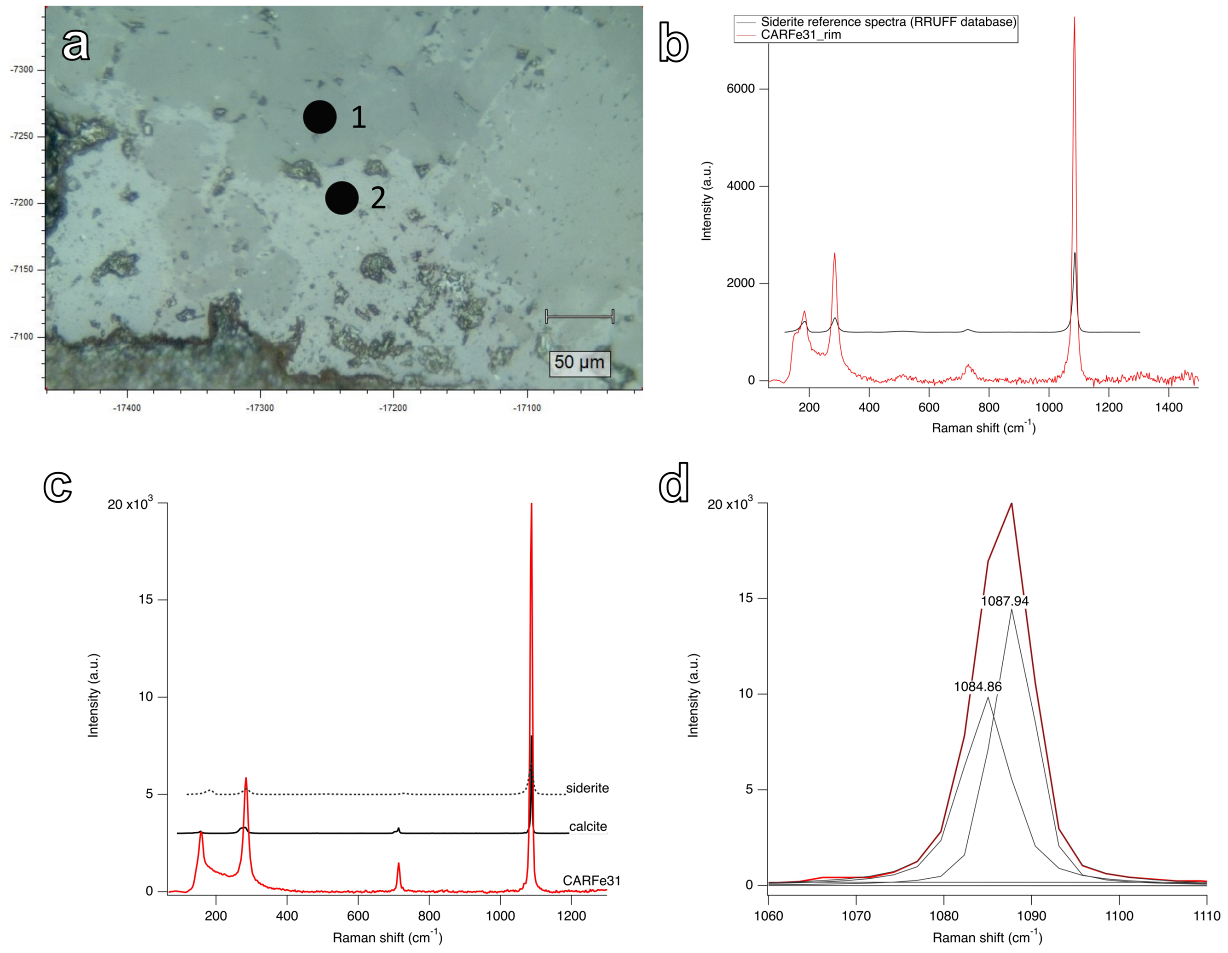

Fig. S 6 Raman characterization of CARFe31. (a) Optical micrograph showing the location of spectra 1, displayed in (b) and spectra 2, displayed in c. Spectra were compared with spectra for siderite and calcite from the RRUFF database. The bright rim consists of siderite, whereas the darker part consists of a siderite/calcite mixture as apparent in the characteristic peak at 1084 cm-1 (siderite) and 1087 cm-1 (calcite) displayed in (d).

9 Grain Boundary Width SEM Image Analysis Details

Scanning electron microscopy (SEM) images were collected at high magnification (Pixel size ~5-10 nm) at 15 kV and recorded using a backscatter detector. Images were first binarized and then clusters were filtered to reduce artificial roughness introduced by binarization. Grain boundary width was analysed using the local thickness function in ImageJ. After analysis, measured widths were normalized by dividing individual values through the sum of all measurements. Afterwards, all measurements below the resolution limit (4 x pixel size) were discarded.

Cleaning scripts:

```
#!/usr/bin/env python3
import sys, os
import numpy as np
from skimage import io
from scipy.ndimage import measurements, morphology, convolve, binary_dilation, binary_erosion
def clean_clusters(bin_image):
    sh = bin_image.shape
    sh = np.asarray(sh)
    sh = sh + 2
    aux = np.ones(shape=(sh))
    aux[1:-1, 1:-1] = bin_image
    lw, num = measurements.label(aux)
    # get a label of the biggest cluster
    minLab = np.min(lw)
    maxLab = np.max(lw)
    print("labels:  min: {}   max: {}".format(minLab, maxLab), flush=True)
    hist = measurements.histogram(lw, minLab + 1, maxLab, maxLab - minLab)
    maxClLab = np.argmax(hist) + 1
    print("label of a biggest cluster: {}".format(maxClLab), flush=True)
    aux[lw != maxClLab] = 0
    return (aux[1:-1, 1:-1]).astype(np.uint8)
arg = sys.argv
data_dir = arg[1]
output_dir = "{}_clean".format(data_dir)
os.mkdir(output_dir)
tif_files = sorted([f for f in os.listdir(data_dir)
                    if (os.path.isfile(os.path.join(data_dir, f)) and ".tif" in f)])
print("Read and scale thickness data from directory: {}".format(data_dir))
for j, filename in enumerate(tif_files):
    print('\n*******************************************')
    print('  Processing file {}'.format(filename))
    print('*******************************************')
    filen = os.path.join(data_dir, filename)
    filename_noext, file_extension = os.path.splitext(filename)
    stack_tif = io.imread(filen, plugin='tifffile')
    print("Tif file dimensions: {}".format(stack_tif.shape))
    stack_tif = clean_clusters(stack_tif)
    stack_tif = binary_dilation(stack_tif).astype(np.uint8)
    stack_tif = binary_erosion(stack_tif).astype(np.uint8)
    stack_tif *= 255
    file_res = os.path.join(output_dir, "{}_cl.tif".format(filename_noext))
    io.imsave(file_res, stack_tif, plugin='tifffile')
```

Normalization script:

```
#!/usr/bin/env python3
import sys, os
import numpy as np
import csv
import pandas as pd
from skimage import io
from scipy.ndimage import measurements, morphology, convolve
import matplotlib.pyplot as plt
arg = sys.argv
data_dir = arg[1]
tif_files = sorted([f for f in os.listdir(data_dir)
                    if (os.path.isfile(os.path.join(data_dir, f)) and ".tif" in f)])
minVal = 1000000.0
maxVal = 0.0
print("Read and scale thickness data from directory: {}".format(data_dir))
for j, filename in enumerate(tif_files):
    print('\n*******************************************')
    print('  Processing file {}'.format(filename))
    print('*******************************************')
    filen = os.path.join(data_dir, filename)
    scale_file = "{}.txt".format(filename[:-11])
    scale_filen = os.path.join(data_dir, scale_file)
    if not os.path.isfile(scale_filen):
```

```
        print("*** Warning. File {} does not exist in  {}  directrory. Skipping this tif. Check
filenames.".format(scale_file, data_dir))
        continue
    else:
        scale = 1.0
        with open(scale_filen) as f:
            for line in f:
                if "PixelSize" in line:
                    scale = float(line.split('=')[1])
                    break
        print(scale)
    stack_tif = io.imread(filen, plugin='tifffile')
    print("Tif file dimensions: {}".format(stack_tif.shape))
    # scaling to the pixel dimensions
    stack_tif = scale * stack_tif
    minVal = min(minVal, np.min(stack_tif))
    maxVal = max(maxVal, np.max(stack_tif))
print("total min: {}  max: {} in real units".format(minVal, maxVal))
numBins = int(maxVal+1)
# numBins = 4 * int(maxVal+1) # this will increase the resolution by dividing bins into 4
hist = np.zeros(numBins, dtype=np.int64)
# now filling the histogram
for j, filename in enumerate(tif_files):
    filen = os.path.join(data_dir, filename)
    scale_file = "{}.txt".format(filename[:-11])
    scale_filen = os.path.join(data_dir, scale_file)
    if not os.path.isfile(scale_filen):
        print("*** Warning. File {} does not exist in  {}  directrory. Skipping this tif. Check
filenames.".format(scale_file, data_dir))
        continue
    else:
        scale = 1.0
        with open(scale_filen) as f:
            for line in f:
                if "PixelSize" in line:
                    scale = float(line.split('=')[1])
                    break
        print(scale)
    stack_tif = io.imread(filen, plugin='tifffile')
    stack_tif[stack_tif<4] = 0
    # scaling to the pixel dimensions
    stack_tif = scale * stack_tif
    stack_tif[stack_tif<20] = 0
    hist = np.add(hist, measurements.histogram(stack_tif, minVal, maxVal, numBins))
a_tot = 0
for di, Ni in enumerate(hist):
    if di>0:
        a_tot += float(Ni)/float(di)
# calculate average diameter
# ignoring hist[0] because all of those are solid pixels
d_av = np.sum(hist[1:])/a_tot
# values of boundary width in pixels
dis = np.linspace(minVal, maxVal, numBins)
# this is a normalized histogram which shows fraction
# of a particular width in the analysed image
# sum over it will give 1 (or 100%)
hist_norm = np.divide(hist[1:],dis[1:])/a_tot
plt.bar(dis[1:], hist_norm, width=25)
plt.ylabel('Fraction')
plt.ylim(0,0.1)
plt.xlim(0,5000)
plt.xlabel('Grain Boundary width [nm]')
plt.savefig('CAR150_20nm_4pixel_fixed.png', dpi=300)
print("average diameter in nm: {}".format(d_av))
# here we should save CSV file with two columns : dis[i], hist_norm
#save information as csv file
#define structure of output
with open (r'output_CARFe59_20nm_4pixel.txt', 'w') as f:
       for i in range(len(hist_norm)):
              f.write("{}   {} \n".format(dis[i+1],hist_norm[i]))
```

10 Additional Information about Replacement Rates Differences

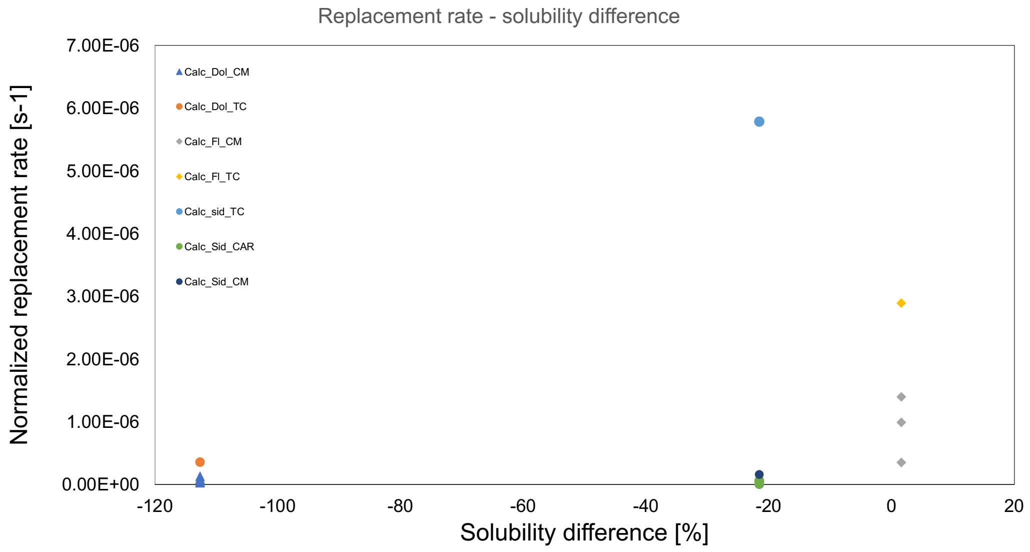

Fig. S 7 Dependence of normalized replacement rates (see main manuscript for calcite-siderite replacement rates, Weber et al., 2021 for calcite-dolomite rates and Weber et al., 2019 for calcite-fluorite rates) on solubility difference between endmembers.

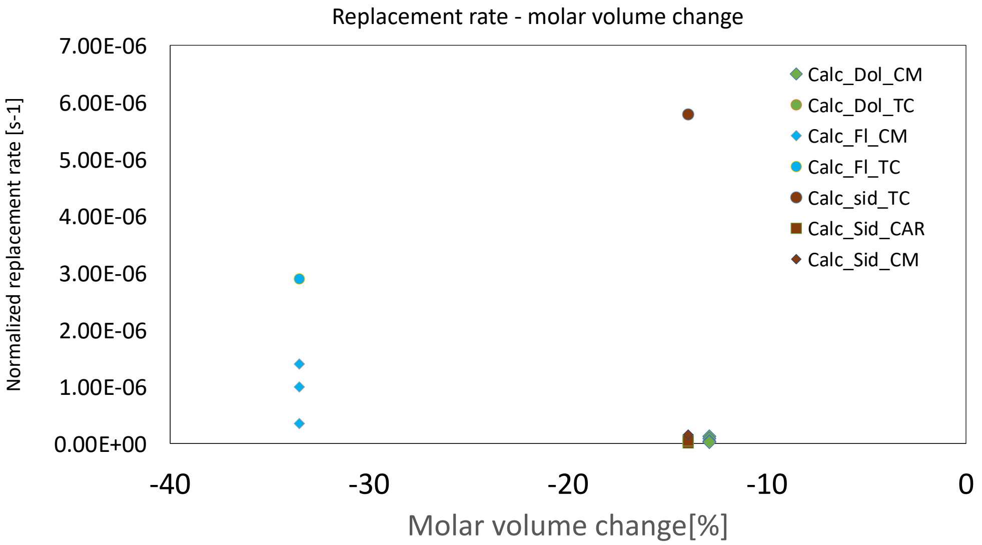

Fig. S 8 Dependence of normalized replacement rates (see main manuscript for calcite-siderite replacement rates, Weber et al., 2021 for calcite-dolomite rates and Weber et al., 2019 for calcite-fluorite rates) on molar volume change between endmembers.

11 Comparison of Grain Boundary Width and Siderite Growth

Table S 1 Comparison between grain boundary width and expected width based on growth rate extrapolation (Jiang and Tosca, 2020) based on V_m = 29.38 cm^3/mol (Robie and Bethke, 1962).

Reaction time [d]	Reaction time [s]	Grain boundary width average [nm]	Grain boundary width maximum [nm]	Siderite grown at SI = 1 [nm]	Siderite grown at SI = 0.6 [nm]	Siderite grown at SI = 0.47 [nm]	Width expected based on growth at SI = 1 [nm]	Width expected based on growth at SI = 0.6 [nm]	Width expected based on growth at SI = 0.47 [nm]
0	0	82	561	0.00	0.00	0.00	82	82	82
14	1209600	496	1817	62.77	23.28	9.97	144.77	105.28	91.97
31	2678400	736	2989	138.98	51.55	22.08	220.98	133.55	104.08
59	5097600	592	1700	264.52	98.12	42.03	346.52	180.12	124.03
120	10368000	163	935	538.00	199.56	85.49	620.00	281.56	167.49

12 Additional SEM and SEM-EDS Data

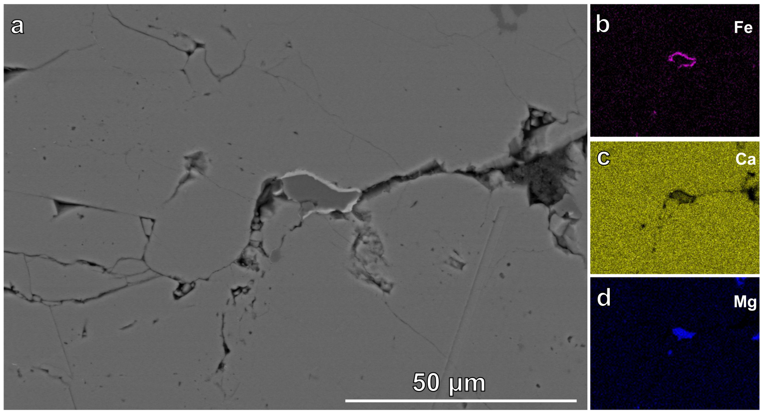

Fig. S 9 Chemical map of CARFe120. (a) SEM-BSE image of siderite layer on dolomite impurity. Elemental maps collected via SEM-EDS of Fe (b), Ca (c) and Mg (d) are also shown.

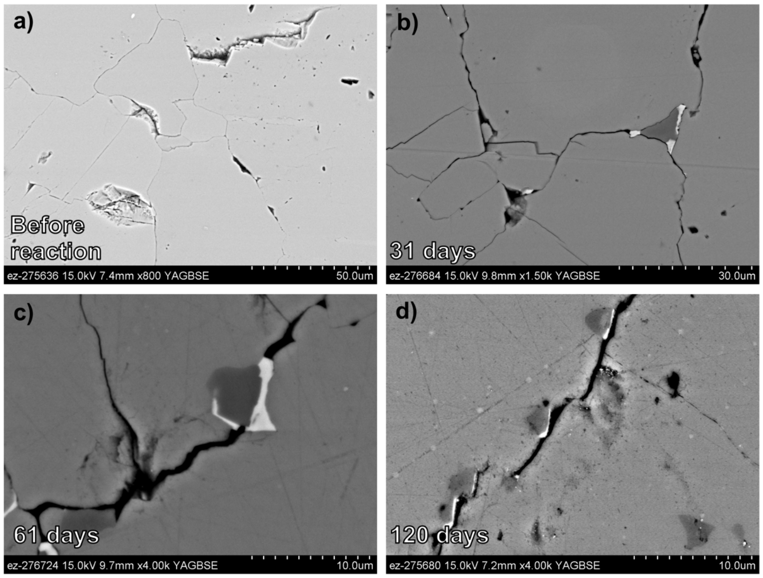

Fig. S 10 SEM-BSE images showing siderite nucleation on dolomite leading to grain boundary widening. Pristine CAR limestone prior to reaction (a), after 32 days of reaction with saturated $FeCl_2$ (b), after 59 days (c) and 120 days (d).

13 References

Jiang C. Z. and Tosca N. J. (2020) Growth kinetics of siderite at 298.15 K and 1 bar. *Geochim. Cosmochim. Acta* **274**, 97–117. Available at: https://doi.org/10.1016/j.gca.2020.01.047.

Robie R. A. and Bethke P. M. (1962) Molar volumes and densities of minerals. *Unites States Dep. Inter. Geol. Surv.*, 4–21.
